# *bb0689* contributes to the virulence of *Borrelia burgdorferi* in a murine model of Lyme disease

**DOI:** 10.1128/iai.00459-24

**Published:** 2024-12-16

**Authors:** Connor Waldron, Sierra George, Christina Thompson, Yu Hsien Liao, Zhiming Ouyang

**Affiliations:** 1 Department of Molecular Medicine, University of South Florida 685045 , Tampa, Florida, USA; Washington State University, Pullman, Washington, USA

**Keywords:** Lyme disease, *Borrelia burgdorferi*, RpoS, virulence regulation, gene regulation, pathogenesis

## Abstract

*Borrelia burgdorferi*, the Lyme disease pathogen, continuously changes its gene expression profile in order to adapt to ticks and mammalian hosts. The alternative sigma factor RpoS plays a central role in borrelial host adaptation. Global transcriptome analyses suggested that more than 100 genes might be regulated by RpoS, but the main part of the regulon remains unexplored. Here, we showed that the expression of *bb0689*, a gene encoding an outer surface lipoprotein with unknown function, was activated by RpoS. By analyzing gene expression using luciferase reporter assays and quantitative reverse transcription PCR, we found that expression of *bb0689* was induced by an elevated temperature, a reduced pH, and increased cell density during *in vitro* cultivation. The transcriptional start site and a functional promoter for gene expression were identified in the 5′ regulatory region of *bb0689*. The promoter was responsive to environmental stimuli and influenced by RpoS. We also showed that *bb0689* expression was expressed in *B. burgdorferi* during animal infection, suggesting the importance of this gene for infection. We further generated a *bb0689* mutant and found that the infectivity of the mutant was severely attenuated in a murine infection model. Although *bb0689*-deficient spirochetes exhibited no defect during *in vitro* growth, they were defective in resistance to osmotic stress. *Cis*-complementation of the mutant with a wild-type copy of *bb0689* fully rescued all phenotypes. Collectively, these results demonstrate that the RpoS-regulated gene *bb0689* is a key contributor to the optimal infection of *B. burgdorferi* in animals.

## INTRODUCTION

*Borrelia burgdorferi* (aka *Borreliella burgdorferi*), the spirochete pathogen causing Lyme disease, is maintained in nature via a complex enzootic life cycle involving *Ixodes* ticks and various vertebrate hosts (usually small rodents) (1–6). Differential gene expression is a major strategy employed by *B. burgdorferi* to adapt to these two drastically different environments. To this end, the spirochete encodes three σ factors, including the major factor, RpoD (σ^70^), and two alternative factors, RpoN (σ^54^) and RpoS (σ^38^). Among these three factors, RpoN and RpoS form a regulatory pathway, where RpoN binds to a –24/–12 promoter and controls the transcription of *rpoS* (7). The RpoN-RpoS pathway (aka the σ^54^-σ^S^ cascade) is activated during tick-mammal transmission and during mammalian infection (6, 8–10). Activation of this pathway requires two regulatory proteins including Rrp2 and BosR. Rrp2 is a homolog of bacterial enhancer-binding proteins (aka AAA+ activator ATPase). Although much remains to be studied about how Rrp2 functions in *B. burgdorferi*, this protein was proposed to be phosphorylated and then functions as an ATPase-providing energy for opening the RpoN-promoter closed complex (11–17). BosR belongs to the Fur family; this protein has also been reported to function as PerR and regulate *B. burgdorferi* oxidative stress response (18–21). How BosR regulates *rpoS* expression is also elusive. BosR has been shown to bind to the *rpoS* promoter region DNA to act as a transcriptional regulator (22–24), and it has also been reported to bind and stabilize *rpoS* mRNA (25).

It has been well documented that the RpoN-RpoS pathway plays a central role in the infection of *B. burgdorferi* in mammalian hosts (6, 10). More specifically, RpoS, as a master regulator, controls expression of many infection-associated outer surface lipoproteins such as outer surface protein C (OspC) and decorin-binding proteins (Dbps) A and B. DbpB and DbpA function as adhesins implicated in the binding of spirochetes to connective tissues (26–28), whereas OspC has pleiotropic roles such as facilitating *B. burgdorferi* invasion of tick salivary glands, binding complement component C4b to inhibit the classical and lectin complement pathways, and promoting *B. burgdorferi* evasion of macrophages (29–32). By analyzing genetic mutants, studies have shown that deletion of the *ospC* gene rendered spirochetes noninfectious, and lack of DbpA and DbpB dramatically reduced borrelial infectivity. Global gene expression analyses have suggested that, in addition to *ospC*, *dbpA*, and *dbpB*, more than 100 other genes may also be regulated by RpoS (8, 33–36). Studies heretofore have been confined to only several RpoS-regulated genes, and the contributions of the main body of the regulon to *B. burgdorferi* infection remain unexplored. Given the importance of the RpoN-RpoS pathway in *B. burgdorferi* mammalian infection, we have focused on elucidating the functions of the lipoproteins influenced by the RpoN-RpoS pathway in order to identify new virulence factors in *B. burgdorferi*. In this study, we showed that *bb0689*, a gene encoding an outer surface lipoprotein with unknown function, is indeed an RpoS-regulated gene, and its expression is induced by several environmental factors. Furthermore, we found that *bb0689* is expressed during animal infection, and its inactivation of *bb0689* significantly attenuates the infectivity potential of *B. burgdorferi*.

## RESULTS

### Analysis of *bb0689* expression under *in vitro* cultivation conditions

**Fig 1:**
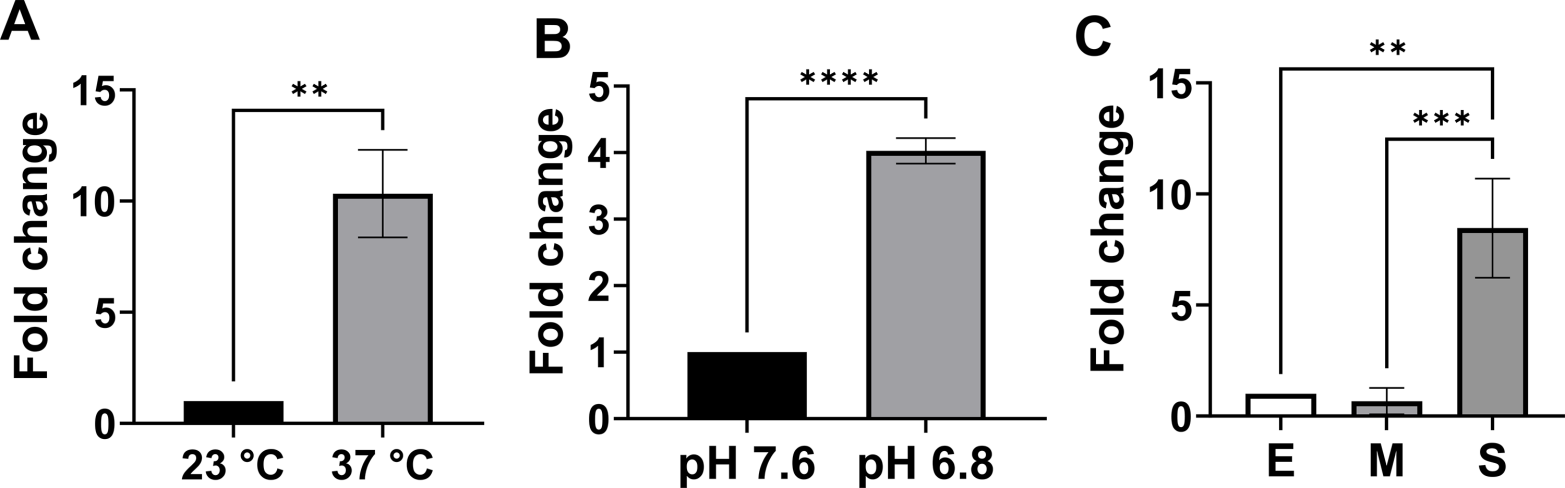
*bb0689* expression is influenced by environmental factors during *in vitro* cultivation. (**A**) *bb0689* expression was induced by elevated temperature. *B. burgdorferi* was cultivated in BSK-II, pH 7.6, at either 23°C or 37°C and was harvested when growth reached the stationary phase. (**B**) *bb0689* expression was induced by lower pH. *B. burgdorferi* was cultured at 37°C in BSK-II with either pH 7.6 or pH 6.8, and spirochetes were collected when bacterial growth reached the stationary phase. (**C**) *bb0689* expression was influenced by cell density or growth phase. *B. burgdorferi* was cultured at 37°C in BSK-II with pH 7.6, and spirochetes were collected when growth reached early logarithmic phase (E), mid-logarithmic phase (M), or stationary phase (S). Gene expression was analyzed by qRT-PCR via relative quantification using *B. burgdorferi flaB* gene as an internal control. All data were collected from three independent experiments, and the bars represent the mean fold changes ± SD. The asterisk indicates statistical significance using one-way ANOVA followed by post-hoc analysis of Tukey (**P < 0.005, ***P < 0.0005, and ****P < 0.00005).

*B. burgdorferi* encounters changes in temperature and pH during its transit between ticks and mammals. *B. burgdorferi* responds to these changes and alters gene expression in order to adapt to different niches (6, 10). As an antibody against BB0689 remains unavailable, the influences of these environmental cues on *bb0689* expression were analyzed by using quantitative reverse transcription PCR (qRT-PCR). To determine the effect of temperature change on gene expression, wild-type (WT) *B. burgdorferi* strain CE162 was cultivated in Barbour-Stoenner-Kelly II (BSK-II) with pH 7.6 at either 23°C or 37°C. Cells were harvested at the stationary phase (~1 × 10^8^ cells/mL), and RNA was isolated and then subjected to qRT-PCR analysis. As shown in Fig. 1A, *bb0689* expression was induced ~10-fold by an elevated temperature. To examine whether pH changes affect gene expression, cells were also harvested from *B. burgdorferi* cultivated in BSK-II with either pH 7.6 or pH 6.8. Our data indicated that *bb0689* expression was upregulated similar to threefold in spirochetes grown at pH 6.8. To assess the effect of cell density and/or growth phase on gene expression, spirochetes were grown at 37°C in BSK-II with pH 7.6, and spirochetes were collected when growth reached the early logarithmic phase (E, ~2 × 10^7^ spirochetes/mL), the mid-logarithmic phase (M, ~5 × 10^7^ spirochetes per mL), and the stationary phase (S, ~1 × 10^8^ spirochetes/mL). Relative to gene expression in spirochetes collected at the early logarithmic phase, *bb0689* expression was not significantly changed in cells at the mid-logarithmic phase but was upregulated similar to sevenfold at the stationary phase. These results are in good agreement with previous global gene profiling results (37, 38).

### Identification of the promoter elements involved in *bb0689* expression

**Fig 2:**
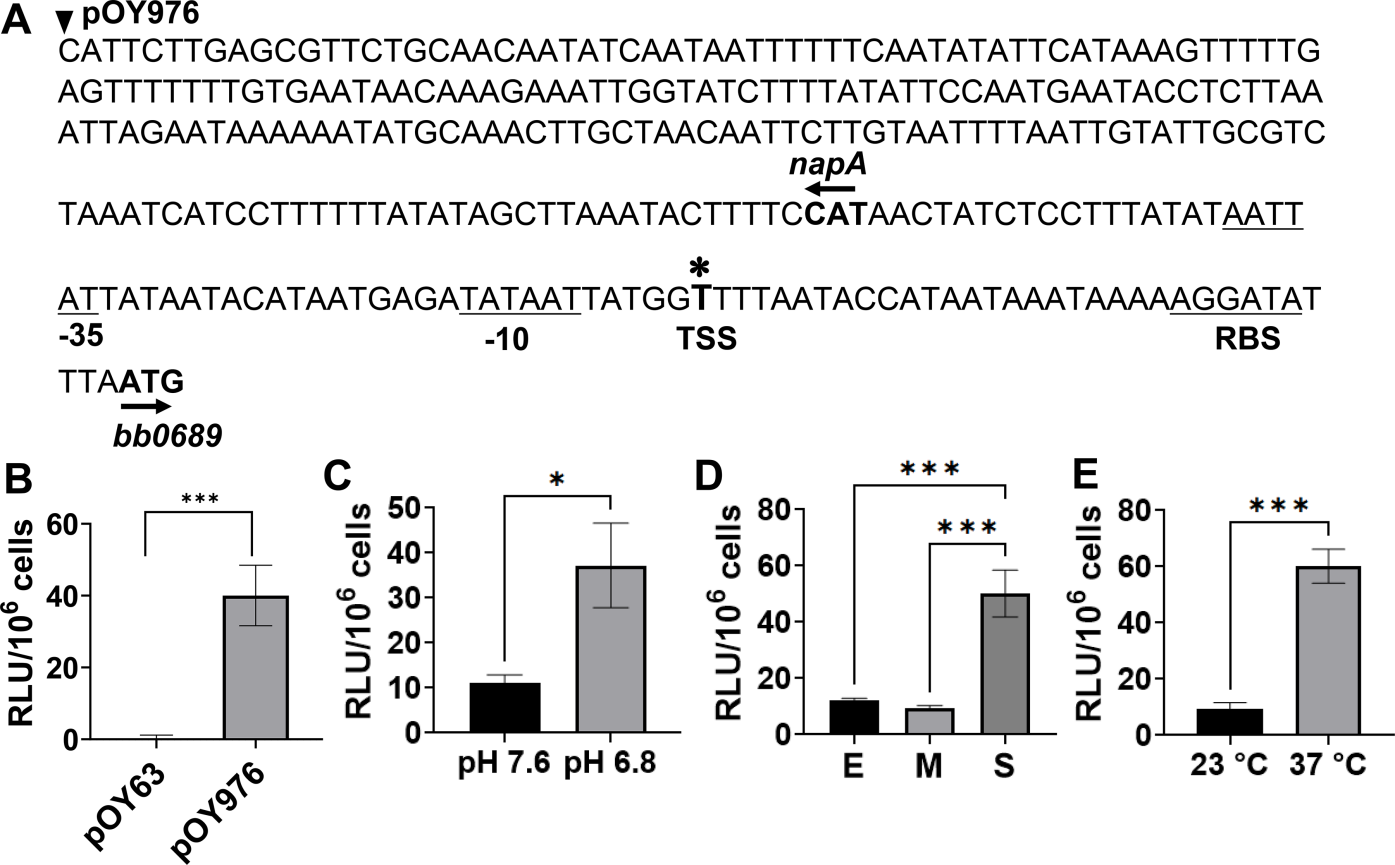
The *bb0689* promoter is responsive to environmental stimuli. (**A**) Schematic diagram of the 5′ regulatory region of *B. burgdorferi bb0689*. The asterisk denotes the TSS identified by 5′ RACE. The associated −35 and −10 elements and the RBS are underlined; the translational start codon (ATG) of *bb0689* and *napA* is in boldface; arrows indicate the direction of translation. The starting position of DNA sequences cloned into the transcriptional reporter construct pOY976 is denoted by filled arrowhead. (**B**) The *bb0689* promoter is functional in *B. burgdorferi*. Luciferase activity (denoted in relative luciferase unit [RLU]/10^6^ cells) detected in *B. burgdorferi* CE162 transformed with various luciferase transcriptional fusion constructs. Spirochetes were cultured in BSK medium at 37°C and harvested at the stationary phase. pOY63, cloning vector containing a promoterless *luc*; pOY976, vector containing the 5′ regulatory region of *bb0689*. (**C–E**) Influence of culture pH (**C**), growth phase (**D**), and temperature (**E**) on luciferase expression in CE162 transformed with pOY976. *B. burgdorferi* was cultivated in BSK medium under specific conditions described in Fig. 1, and luciferase activity (denoted in RLU/10^6^ cells) was measured. In B, C, D, and E, results from three independent experiments are presented as the mean values ± SD. Statistical significance was determined by using an unpaired Student’s *t* test (**B, C, and E**) or ANOVA followed by a Tukey’s test (**D**). The asterisk indicates statistical significance (*P < 0.05 and ***P < 0.0005).

To define the *bb0689* transcriptional unit and the promoter for gene expression, we first mapped the transcriptional start site (TSS) using rapid amplification of 5′ cDNA ends (5′ RACE). RNA was extracted from *B. burgdorferi*, and cDNA was synthesized using gene-specific primers. A poly-C tail was added to cDNA to facilitate further amplification and cloning. The PCR product was then cloned and analyzed via DNA sequencing. Based on the sequencing results from fifteen clones, the *bb0689* TSS was mapped to a single thymine nucleotide 33 bp upstream of the ATG start codon of *bb0689* (Fig. 2A). This result is consistent with the previous 5′ end RNA-seq result described by Adams et al. (39). Based on this information, a putative bacterial σ^70^ promoter (Fig. 2A) was identified in the 5′ regulatory region of *bb0689*. Specifically, the −10 site consists of an AT-rich sequence (TATAAT) located 39–44 bases upstream of the ATG start codon, whereas the −35 site consists of a sequence of AATTAT located at 62–67 bases upstream of the start codon. A putative ribosomal-binding site (RBS; AGGATA) was predicted in a region 5–10 bp upstream of the start codon. To determine whether this promoter is active in *B. burgdorferi*, we conducted luciferase reporter assays. Specifically, the 5′ regulatory region encompassing the putative promoter elements was amplified from WT and cloned into the promoterless *luc* reporter vector pOY63, allowing the cloned DNA to be fused with *luc*. This strategy places the transcription of *luc* under the direct control of the cloned DNA. The resultant luciferase transcriptional fusion construct pOY976 was introduced into strain CE162, yielding strain OY634. This strain was then grown in BSK-II at 37°C. Cells were harvested when growth reached the stationary phase and subjected to luciferase activity analysis. As shown in Fig. 2B, luciferase was detected in the strain harboring pOY976 but not from the strain harboring the empty vector pOY63. These data suggest that the putative promoter is functional and drives *bb0689* expression in *B. burgdorferi*.

### The *bb0689* promoter is responsive to environmental stimuli

Our qRT-PCR analysis revealed that *bb0689* expression is influenced by various environmental parameters such as temperature, pH, and cell density (Fig. 1). To explore whether these factors impact *bb0689* expression through the promoter located in the 5′ regulatory region, luciferase activities were measured in WT *B. burgdorferi* harboring pOY976 under various environmental conditions. As shown in Fig. 2C, luciferase expression was induced in spirochetes grown at pH 6.8 (vs pH 7.6). Moreover, *B. burgdorferi* was cultivated at pH 7.6 and collected at different growth phases. Relative to luciferase activity in spirochetes at the early logarithmic phase, luciferase expression was not significantly changed in the mid-logarithmic phase cells but markedly induced in cells collected at the stationary phase (Fig. 2D). We also examined the effect of temperature on *bb0689* expression using the luciferase reporter assays. Relative to gene expression in spirochetes grown at 23°C, luciferase expression was significantly increased in spirochetes cultured at 37°C (Fig. 2E). These data are consistent with our qRT-PCR data (Fig. 1) and support that the environmental factors influence *bb0689* expression through the promoter.

### *bb0689* expression is regulated by RpoS in *B. burgdorferi*

**Fig 3:**
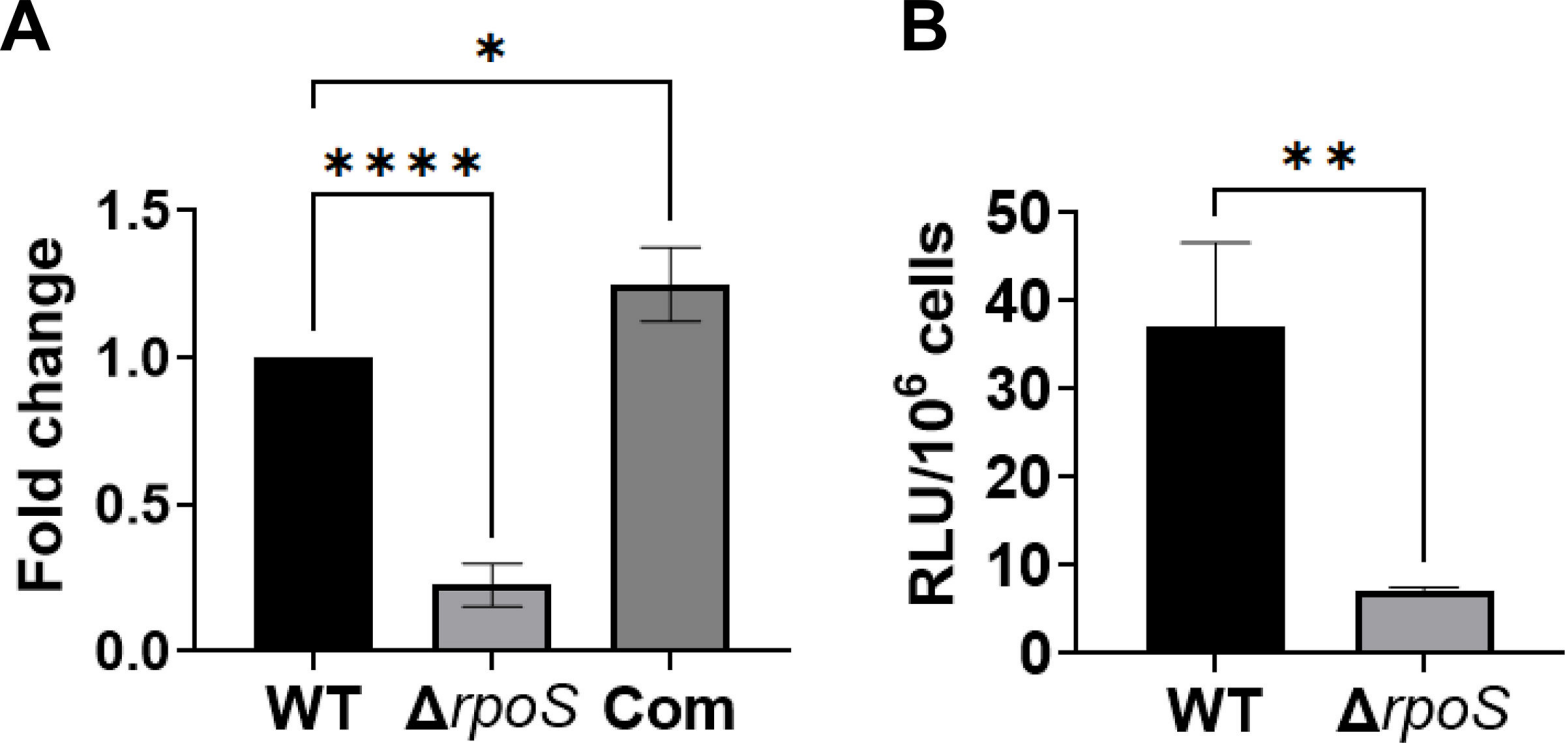
*bb0689* expression is regulated by RpoS in *B. burgdorferi*. (**A**) *bb0689* expression among strain 297 (WT), the *rpoS* mutant BbJSB19-A7B (Δ*rpoS*), and the complemented strain (Com) were compared using qRT-PCR via relative quantification. Data were normalized by using *B. burgdorferi flaB* gene as an internal control. (**B**) Activity of the *bb0689* promoter is regulated by RpoS. Once the transcriptional reporter construct pOY976 was introduced into WT CE162 or the *rpoS* mutant OY517 (Δ*rpoS*), the resultant strains were cultured in BSK medium at 37°C and harvested at the stationary phase for luciferase activity measurement. Results from three independent experiments are indicated as the mean values ± SD. Statistical significance was determined by using ANOVA followed by a Tukey’s test (**A**) or an unpaired Student’s *t* test (**B**). The asterisk indicates a statistically significant difference (*P < 0.05, **P < 0.005, and ****P < 0.00005).

The alternative sigma factor RpoS is a central regulator that modulates differential gene expression in *B. burgdorferi*. Previous global gene expression analyses by us and others suggested that *bb0689* expression may be regulated by RpoS (8, 33–36). To garner more direct evidence for the role of RpoS in *bb0689* expression, a *rpoS* mutant BbJSB19-A7B (36), its isogenic complemented strain, and the parental strain 297 were cultivated in BSK-II to stationary phase at 37°C. RNA was isolated from these spirochetes and subjected to qRT-PCR analyses. As shown in Fig. 3A, *bb0689* expression was significantly downregulated in the *rpoS* mutant and restored to a WT level in the complemented strain.

We further determined whether RpoS regulates gene expression through the *bb0689* promoter. Based on sequence information, the *bb0689* promoter is a σ^70^-type promoter; this promoter or the upstream 5′ regulatory region of *bb0689* does not share significant sequence similarity with RpoS-dependent promoters of *ospC* or *dbpBA*. To determine whether the activity of *bb0689* promoter is regulated by RpoS, we introduced the luciferase reporter construct pOY976 into a *rpoS*-deficient strain and measured the luciferase activity in the resultant strain OY650. Relative to gene expression in WT strain OY634, luciferase expression from pOY976 was significantly lower in the *rpoS* mutant (i.e., strain OY650; Fig. 3B). These combined data support that RpoS regulates *bb0689* expression via the *bb0689* promoter.

### *bb0689* is expressed during animal infection

**Fig 4:**
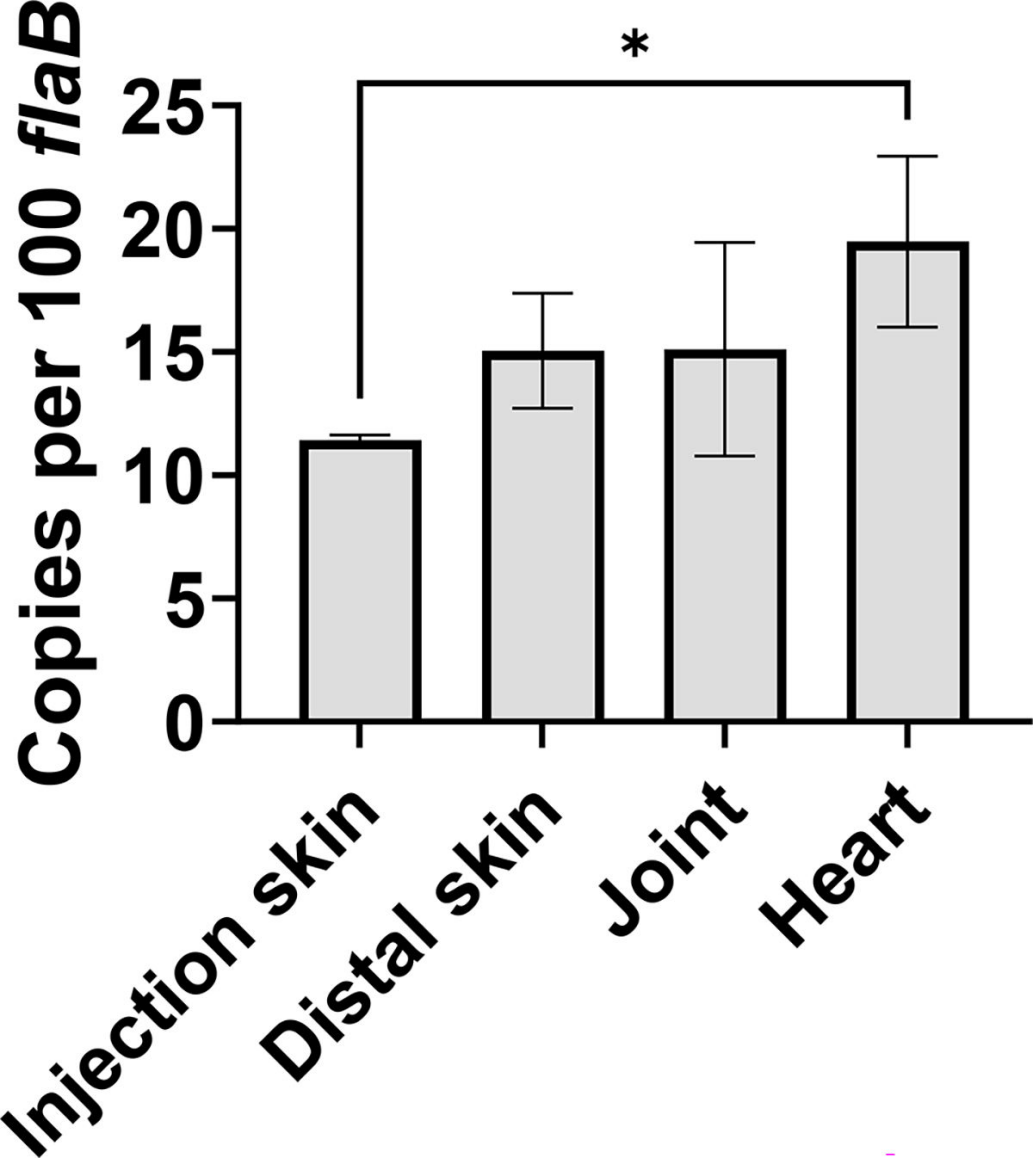
*bb0689* is expressed by *B. burgdorferi* during animal infection. C3H mice were infected with *B. burgdorferi* CE162 via intradermal injection and sacrificed at day 11 postinfection. Samples, including injection skin, and distal skin, heart, and joints, were collected. RNA was isolated, and copies of *B. burgdorferi flaB* and *bb0689* were measured via absolute quantification qRT-PCR using the standard curve method. Data were collected from six infected mice, and the bars represent the mean values ± SD. The values represent the average copy number of *bb0689* normalized per 100 copies of *flaB* transcripts. Asterisks indicate statistical significance using ANOVA followed by Tukey test (*P < 0.05).

Given that *bb0689* is differentially expressed, we examined *bb0689* expression in infected animals via qRT-PCR analyses. C3H mice were infected with WT CE162 via intradermal injection. Infected animals were euthanized, and tissue samples, including heart, joint, skin from the injection site (i.e., injection skin), and skin from the distal back (i.e., distal skin), were collected. Total RNA was extracted, and copy numbers of *B. burgdorferi bb0689* and *flaB* were measured by qRT-PCR via absolute quantification. As shown in Fig. 4, *bb0689* was expressed at comparable levels in all animal tissues. Approximately 10–20 copies of *bb0689* transcripts per 100 *flaB* transcripts were detected in the samples. These results suggest that the *bb0689* gene is expressed in spirochetes, while they are replicating in the mammalian host. This observation is consistent with the notion that *bb0689* expression is influenced by the RpoN-RpoS pathway, the pathway that is active during mammalian infection.

### Construction of the *bb0689* mutant and complemented strains

**Fig 5:**
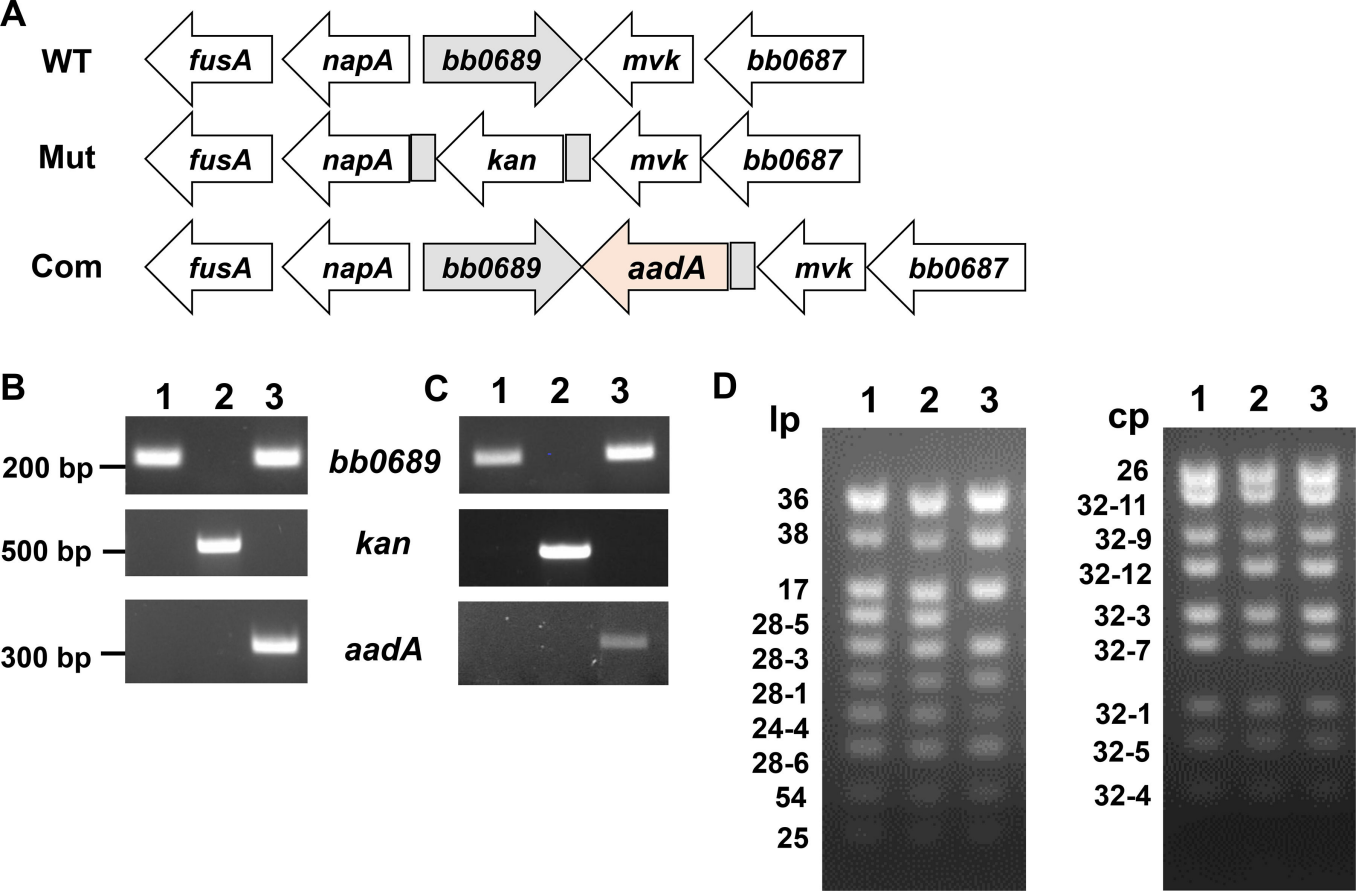
Construction of *bb0689* deletion mutant and its isogenic complemented strain. (**A**) Schematic representation (not drawn to scale) of the *bb0689* locus in WT strain OY444, the mutant OY470 (Mut), and the relevant complemented strain OY601 (Com). Genes are shown as thick arrows circumscribing the respective gene numbers. PCR (**B**) and reverse transcription-PCR (RT-PCR) (**C**) analyses of WT OY444, the *bb0689* mutant, and the complemented strain. DNA sizes are indicated in base pairs (bp) on the left. (**D**) Plasmid content of *B. burgdorferi* was determined using multiplex PCR. lp, linear plasmid; cp, circular plasmid. Lane 1, WT OY444; lane 2, the *bb0689* mutant; lane 3, the complemented strain.

To investigate the role of *bb0689* in borrelial infection, we generated a *bb0689*-deficient mutant by using the allele exchange method. Upon introducing the suicide vector pOY43 into strain OY444, the infectious bioluminescent strain derived from CE162, the chromosomal *bb0689* was disrupted with the PflgB-kan cassette, while the surrounding region remains intact (Fig. 5A). To complement *bb0689* mutation in *cis*-, we generated another suicide vector pOY928 that carries an intact *bb0689* and then transformed this vector into the *bb0689* mutant. Through homologous recombination, *bb0689* was restored at its original locus, and a PflgB-aadA cassette was inserted just downstream of *bb0689* (Fig. 5A). The mutant and complemented strain were analyzed by PCR. A fragment was successfully amplified from WT OY444 and the complement, but not from the mutant, using *bb0689*-specific primers (Fig. 5B). In contrast, by using kanamycin-specific primers, a fragment was amplified only from the mutant. A fragment was amplified only from the complement using *aadA*-specific primers (Fig. 5B). The lack of *bb0689* expression in the mutant was confirmed at the RNA level. As expected, *bb0689* transcripts were detected in both WT and the complement, but not in the mutant (Fig. 5C). Kanamycin and *aadA* transcripts were detected only in the mutant or complement, respectively. Further PCR-based plasmid profiling showed that the *bb0689* mutant contained all endogenous plasmids present in the parental strain, and the complemented strain lost only the nonessential linear plasmid lp28-5 but retained all other plasmids including essential plasmids lp28-1 and lp25 (Fig. 5D).

### The *bb0689* mutant was attenuated in its ability to infect mice

**Fig 6:**
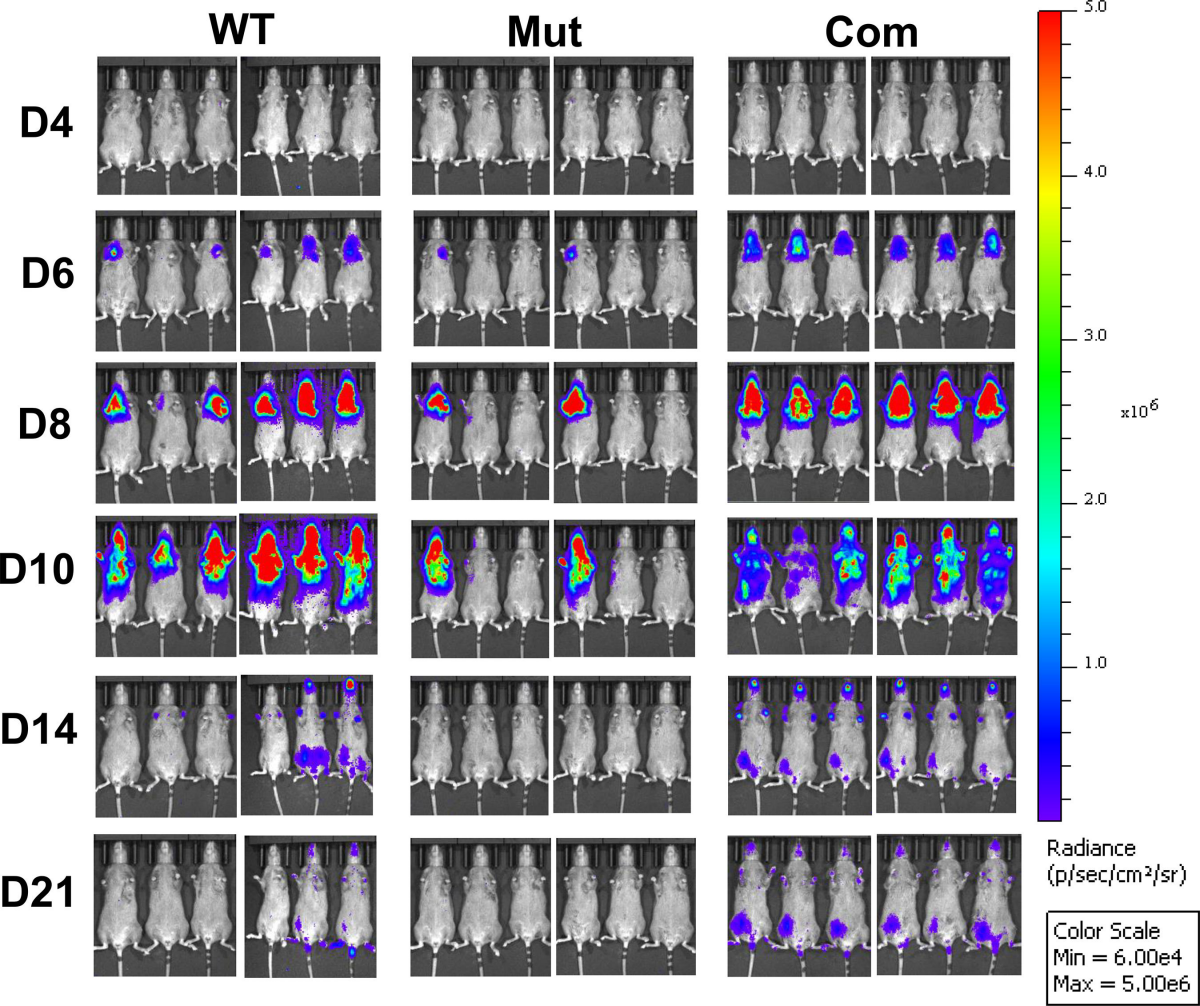
Bioluminescence imaging of *B. burgdorferi* in living mice. Groups of C3H mice were injected intradermally with OY444 (WT), the *bb0689* mutant OY470 (Mut), or the complemented strain OY601 (Com) at a dose of 10,000 spirochetes per mouse. At various time points post infection, mice were injected with D-luciferin and analyzed using a Perkin Elmer IVIS imaging system. To obtain a global assessment of disseminated infection, all animals were consistently exposed for 5 min. All images are normalized to the same p/s range and displayed on the same color spectrum scale (right). D, day.

To assess the contribution of *bb0689* to the temporal and spatial dynamics of *B. burgdorferi* infection, C3H mice were infected intradermally with WT OY444, the *bb0689* mutant, or the complemented strain. The infection was monitored using *in vivo* bioluminescence imaging for 3 weeks. In the group infected with WT OY444, luminesce, as expected, was detected in all six mice starting from Day 6 postinfection, indicating that all mice were infected (Fig. 6). On day 10, signals reached a peak corresponding to the highest number of live spirochetes. From day 10, signals were found to disseminate to the head and tail (Fig. 6). Moreover, the intensity of the luminesce decreased gradually from days 10 to 21 postinfection, probably due to partial clearance of the infection by host immunity. These data are consistent with previous observations (40–42). For the group of mice injected with the complemented strain, luminesces were detected in all six mice and showed a similar pattern to those observed in WT-infected mice (Fig. 6). Surprisingly, for the group of six mice injected with the *bb0689* mutant, luminesce signals were only detected in two mice (Fig. 6).

To substantiate the imaging data, *B. burgdorferi* infection was further determined using the cultivation method. Specifically, mice were sacrificed on day 21 postinfection, and tissue samples were collected and cultured in medium for spirochete growth. For the group of mice infected with WT or the complemented strain, motile spirochetes were recovered from all tissue samples including injection skin, distal skin, joints, and heart (Table 1). In contrast, spirochetes were reisolated from only 8 out of 24 samples collected from mice injected with the *bb0689* mutant; these eight samples, more specifically, were collected from two mice shown to be positive for luminesce imaging. Taken together, these data suggest that *bb0689* is needed for optimal infectivity of *B. burgdorferi* during experimental infection.

**TABLE 1:** Infectivity of *B. burgdorferi* in mice^*a*^

Strain	Dose	No. of cultures positive/total no. of specimens examined					No. of mice infected/total no. of mice
		IS	DS	Joint	Heart	All sites	
WT	10^4^	6/6	6/6	6/6	6/6	24/24	6/6
Mut	10^4^	2/6	2/6	2/6	2/6	8/24	2/6
Com	10^4^	6/6	6/6	6/6	6/6	24/24	6/6

^*a*^ C3H mice were intradermally injected with WT OY444, the *bb0689* mutant OY470 (Mut), or the complemented strain OY601 (Com). Mice were sacrificed at 3 weeks post-inoculation; injection skin (IS), distal skin (DS), heart, and joints were harvested for spirochete recovery in BSK-II.

### *bb0689* is dispensable for *B. burgdorferi* growth in the medium

**Fig 7:**
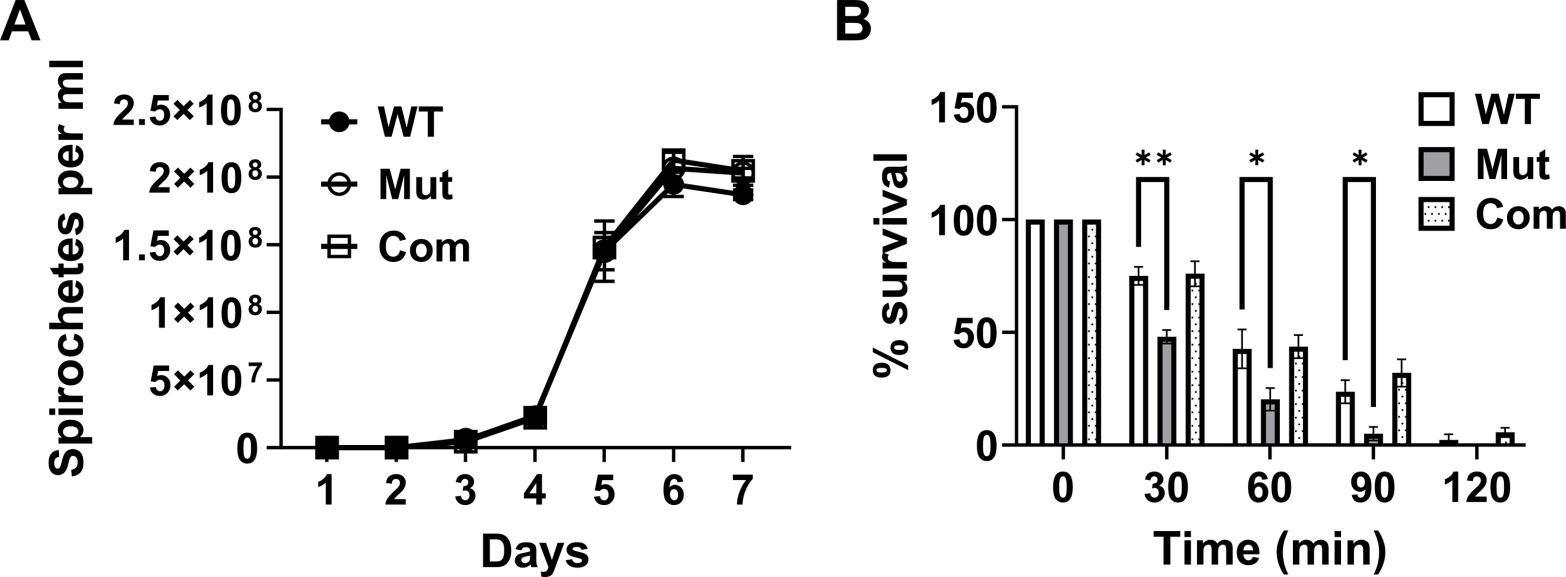
*bb0689* is dispensable for *in vitro* growth but participates in the resistance of *B. burgdorferi* to osmotic stress. (**A**) Growth analysis. To determine growth phenotypes, WT, the *bb0689* mutant (Mut), and the complemented strain (Com) were inoculated to BSK-II, grown at 37°C, and enumerated daily by dark field microscopy. Data are presented as the mean values ± SD from three biological replicates. (**B**) Osmotic shock assays. WT OY444, the mutant, and the complemented strain were grown in BSK-II at 37°C. Once growth reached the mid-logarithmic phase, cultures were exposed to 1 M NaCl for 0, 30, 60, 90, or 120 min. Spirochete viability was determined, and percent survival was calculated. Data are presented as the mean values ± SD from three biological replicates. Asterisks indicate statistical significance using one-way ANOVA followed by Tukey test (*P < 0.05 and **P < 0.005).

To assess the role of *bb0689* in the growth of *B. burgdorferi*, WT OY444, the mutant, and the complemented strain were cultivated in BSK-II medium at 37°C. As shown in Fig. 7A, the *bb0689* mutant exhibited similar growth kinetics to WT OY444 and the complemented strain. With an initial inoculum of 10,000 spirochetes/mL, all strains reached the stationary growth phase at day 6 post inoculation, with a maximum cell density of ~2 × 10^8^ spirochetes/mL. In addition, no obvious differences were observed among WT, the mutant, and the complemented strain when spirochete morphology was examined using dark-field microscopy.

### The *bb0689* mutant is more sensitive to osmotic stress

BB0689 has been reported to be an outer membrane protein in *B. burgdorferi* (43, 44). Such protein may be critical for membrane stability, and its loss may compromise membrane integrity, rendering *B. burgdorferi* more sensitive to osmotic pressure. To assess the role of *bb0689* in resistance to osmotic stress, we compared the survival of WT OY444, the *bb0689* mutant, and the complement upon exposure to 1 M NaCl. Spirochetes died gradually after NaCl was added to the medium, and nearly all spirochetes died when treated with NaCl for 120 min (Fig. 7B). When spirochetes were exposed to NaCl for 30, 60- or 90 min, ~75%, ~45%, and ~25%, respectively, of WT spirochetes survived the treatment (Fig. 7B). The complemented strain survived to the same extent as WT. However, the *bb0689* mutant was more susceptible to 1 M NaCl than WT and the complement. Specifically, ~50%, ~20%, and ~5% of the *bb0689*-deficient spirochetes survived when treated with NaCl for 30, 60, or 90 min, respectively (Fig. 5B). These results indicate that *bb0689* participates in osmotic resistance in *B. burgdorferi*.

## DISCUSSION

RpoS is essential for *B. burgdorferi* mammalian infection. It has been established that this alternative sigma factor plays a central role in *B. burgdorferi* virulence gene expression. To date, several RpoS-regulated virulence factors have been identified, including OspC, DbpB, DbpA, BBK32, BBA07, OppA5, and BB0563 (26–28, 31–33, 40, 41, 45–48). Here, we showed that *bb0689* is another RpoS-regulated virulence gene that contributes to the infection of *B. burgdorferi* in animals, likely by playing an important role in combating osmotic stress.

By analyzing differential gene expression in *B. burgdorferi* cultivated in the medium, we found that *bb0689* expression was induced by elevated temperature, reduced pH, and increased cell density. These results are in good agreement with the previous study by Revel et al. (38). Furthermore, this pattern of *bb0689* expression *in vitro* correlated well with the expression pattern of *rpoS*. Previous global gene expression analyses also suggested that *bb0689* expression may be regulated by RpoS (8, 33–36). This hypothesis was addressed in the current study. qRT-PCR analyses showed that *bb0689* expression was downregulated in the *rpoS* mutant and restored in the complemented strain. Additionally, a promoter for *bb0689* expression was identified in the 5′ regulatory region upstream of *bb0689*. By using promoter-*luc* transcriptional fusion reporter assays, we found that, relative to gene expression in WT strain, luciferase expression was drastically reduced in the *rpoS* mutant. These results clearly indicate that *bb0689* is another gene regulated by RpoS. To date, it remains unknown whether RpoS regulates *bb0689* expression directly or indirectly. Despite multiple attempts, we failed to produce *bb0689* RNA using the *in vitro* transcription system developed by Boyle et al. (49). However, promoter mapping in this study revealed in the sequences upstream of *bb0689* a σ^70^-type promoter that drives gene expression, suggesting that RpoS may compete with RpoD for this promoter. This notion is supported by several lines of evidence. First, RpoS and RpoD recognize nearly indistinguishable promoter DNA (50, 51). Second, *bb0689* showed an expression pattern similar to *rpoS* during *in vitro* cultivation and during animal infection. Finally, unlike other RpoS-dependent genes like *ospC* and *dbpBA*, *bb0689* expression was downregulated, but not abolished, in the *rpoS* mutant.

We also provide direct evidence that *bb0689* is required for optimal infectivity of *B. burgdorferi. bb0689* Transcripts were expressed in infected mice tissues such as skin, joints, and heart, suggesting a role of this gene in borrelial infection. Moreover, when the infectivity of a mutant deficient in *bb0689* was analyzed using two complementary methods, our results revealed that the infectivity of the *bb0689* mutant was attenuated. When gene mutation was complemented in *cis*-, the infectivity was restored to WT levels. These data clearly demonstrate *bb0689* as another virulence gene that contributes to *B. burgdorferi* infection. Of note, the results shown in the current study were obtained when each animal was injected with 10,000 spirochetes. The effect of gene mutation on borrelial infectivity may also be examined in the future using mice receiving other doses of spirochetes (e.g., 100, 1,000, or 100,000 spirochetes per animal). BB0689 was annotated as a lipoprotein with an unknown function (52). Based on protein structure analysis, this protein was recently reported to belong to the CAP (the cysteine-rich secretory proteins, antigen 5, and pathogenesis-related 1 proteins) superfamily (53). The CAP superfamily proteins have been reported in numerous organisms across prokaryotes and eukaryotes. These proteins are involved in a wide variety of biological processes, such as the regulation of extracellular matrix and branching morphogenesis, immune defense in mammals and plants, cell-cell adhesion during fertilization, tumor development, and pathogen virulence (54, 55). Moreover, CAP proteins act as virulence determinants in several fungal pathogens, probably related to their activities in binding/sequestering of sterols and/or related small hydrophobic compounds (55). For instance, sterol sequestration from the host tissue by fungal pathogens might facilitate tissue penetration. However, when such activities were examined for BB0689, no interaction was observed between BB0689 and cholesterol, fatty acids, phosphatidylinositol, or heparan sulfate (53). Thus, BB0689 may impact the pathogenic potential of *B. burgdorferi* via other unique means. BB0689 was reported to be localized at the outer leaflet of the outer membrane (43, 44). Given that BB0689 lacks sequence or structure similarity to typical bacterial adhesins, it appears unlikely for this protein to function as an adhesin to interact with host tissues. Rather, BB0689 may, directly or indirectly, be required for the stability of *B. burgdorferi* cell surface architecture. Compared with the membrane structure of other dual-membrane bacteria, the outer membrane of *B. burgdorferi* is relatively fragile (56, 57). It is possible that loss of BB0689 may compromise outer membrane integrity, which, in turn, impairs borrelial survival under stresses. This notion is supported by our current study demonstrating that the *bb0689* mutant was more susceptible to osmotic stresses than WT bacteria. It is also possible that BB0689, as a part of stress response pathway(s), impacts stress signaling and virulence gene expression in *B. burgdorferi*. Additionally, loss of BB0689 may compromise the expression, localization, and/or the activities of other surface proteins engaging in pathogen-host interactions. These possibilities will be explored in the future to decipher how BB0689 functions as a novel virulence factor.

In this study, we showed that another RpoS-regulated gene *bb0689*, as a novel virulence gene encoding an outer surface lipoprotein, contributes to the optimal infection of *B. burgdorferi* in animals. Inactivation of *bb0689* compromises the capability of *B. burgdorferi* to infect mice. Furthermore, the mutant was much more susceptible than WT and the complement to osmotic stress. Although much remains unknown about how BB0689 impacts *B. burgdorferi* stress response and virulence, this work provides important insights into the relationship between the RpoS regulon and the virulence mechanisms of *B. burgdorferi*. Additionally, it remains possible that BB0689 plays a role during tick infection, although RpoS is not required by *B. burgdorferi* to colonize, survive, and persist in ticks. Therefore, future work may help to address whether the *bb0689* mutant has defects during tick acquisition, survival through the intermolt phases, and tick-mammal transmission. Structure analysis suggested that BB0689 belongs to the CAP protein family; however, functional studies indicated that BB0689 does not share common functions with other CAP proteins (53). Instead, BB0689 may have unique features that are important for *B. burgdorferi* biology and infectivity. Further studies are thus warranted to elucidate the physiological function of this surface lipoprotein.

## MATERIALS AND METHODS

### Bacterial strains and culture conditions

The bacterial strains and plasmids used in this study are listed in Table 2. Infectious *B. burgdorferi* clonal strain CE162 (58) and its bioluminescent derivative OY444 (41) were used as the WT strains throughout this study. The mutant deficient in *bb0689* and its isogenic complemented counterpart were generated using allelic exchange. The endogenous plasmid content of *B. burgdorferi* strains was monitored using multiplex PCR as previously described (59, 60). *B. burgdorferi* was routinely cultivated at 37°C and 5% CO_2_ in BSK-II medium (61), pH 7.6, supplemented with 6% rabbit serum (Pel-Freez Biologicals) and appropriate antibiotics (kanamycin, 160 µg/mL; streptomycin, 50 µg/mL; or gentamycin, 40 µg/mL). The effects of environmental factors, such as temperature, pH, and cell density or growth phase on gene expression, were examined as described previously (38, 41, 58, 62–65). Spirochetes were enumerated by dark-field microscopy with Petroff-Hausser counting chambers (Hausser Scientific). Growth curves of *B. burgdorferi* strains were calculated as previously described (41, 62–64). *Escherichia coli* strain TOP10 was grown at 37°C in Luria-Bertani (LB) broth or on LB plates with appropriate antibiotics (ampicillin, 100 µg/mL; kanamycin, 50 µg/mL; spectinomycin, 100 µg/mL). All recombinant plasmid constructs were confirmed by PCR, restriction digestion, and Sanger sequencing (Azenta Life Science).

**TABLE 2:** Strains and plasmids used in this study

Strain or plasmid	Description	Source
*B. burgdorferi* strains		
297	Virulent *B. burgdorferi*	(66)
BbJSB19-A7B	*rpoS* mutant derived from strain 297	(36)
*rpoS^−^*/pJSB259	*rpoS* complemented strain; BbJSB19-A7B transformed with pJSB259	(36)
CE162	Infectious clonal derivative of strain 297	(58)
OY444	CE162, PflaB-*luc* inserted in the *bbb20-bbb21* intergenic region	(41)
OY470	*bb0689* mutant; OY444 transformed with pOY43	This study
OY517	*rpoS* mutant; CE162 transformed with pOY212	(42)
OY601	*bb0689* complement; OY470 transformed with pOY928	This study
OY634	CE162 transformed with pOY976	This study
OY650	OY517 transformed with pOY976	This study
*E. coli* strains		
Top10	F^−^ *mcrA Δ(mrr-hsdRMS-mcrBC) f80lacZΔM15 ΔlacX74 recA1 araD139 Δ(ara-leu)7697 galU galK rpsL* *(Str^R^) endA1 nupG*	Thermo Fisher Scientific
Plasmids		
pGEM-Teasy	PCR cloning vector	Promega
pOY04	Plasmid containing the PflgB-kan cassette	(67)
pOY40	Overlap-PCR product amplified using primer pair 31.2F/32R cloned into pGEM-Teasy	This study
pOY43	PflgB-Kan cloned into pOY40 at AscI	This study
pOY63	Shuttle vector containing a promoterless *luc*	(68)
pOY928	Suicide plasmid to complement *bb0689* mutation; contains PCR product amplified using primer pair 1251F/1251R, PCR product amplified using primer pair 1252F/1252R, the pUC Ori, and PflgB-aadA cassette	This study
pOY976	PCR product amplified using primer pair 1334F/1334R cloned into pOY63 at NcoI and NdeI sites	This study
pOY16	*flaB* cloned into pGEM-Teasy	(9)
pOY707	Mouse β-actin gene cloned into pSC-B-amp/kan	(69)

### Construction of a *bb0689* mutant in *B. burgdorferi*

To create a suicide vector for generating the *bb0689* mutant by homologous recombination, the 908 bp upstream region and the 1,281 bp downstream region of *bb0689* were PCR amplified from strain OY444 with primer pairs 32F/32R and 31.2F/31R (Table 3), respectively. The DNA fragments were used as templates in an overlap PCR using primer pair 31.2F/32R, and the resultant product was cloned into the pGEM-Teasy cloning vector (Thermo Fisher Scientific), generating pOY40. Next, the kanamycin-resistant cassette PflgB-Kan, excised from pOY04 (67) using AscI, was ligated into pOY40 at the AscI site. In the resultant construct pOY43, the PflgB-Kan cassette was oriented in the opposite direction from transcription of *bb0689*. Purified pOY43 plasmid DNA was then transformed into *B. burgdorferi* strain OY444 using a previously described protocol (70). Kanamycin-resistant transformants were analyzed by PCR and reverse transcription-PCR (RT-PCR) to confirm the loss of *bb0689*.

**TABLE 3:** Oligonucleotide primers used in this study^*a*^

Primer	Sequence (5′−3′)
31.2F	GCGCTTAAAGCTTTGGGAGCCGGAAACG
31R	GGCGCGCCGCATGGCTTAATAGTCCAAGCCACA
32F	GGCGCGCCGCATTGTACTTAAATTGCATGCTTGAG
32R	TTAGCGCGCTTTGCTCTTGAGGCATCCAGTCAGT
260F	CGGCTTGCGTTGCATCAATACTGGAC
260R	TAATGGCGCGCCGCATTGCCTCTTTGTATTTGTCCTGC
261F	TAATGGCGCGCCAGATATAACCTGGACAATAGTCCCAAA
261R	GCTACGCAACAGTCGTCACAAGAGGT
496F	GCACAGCACAATAGAAATAG
496R	TAATGGCGCGCCTTAATTTATTTCTTCTTTTAA
497F	TAATGGCGCGCCCTGAAATTACCCTTGAACAAGAT
497R	TTAGCGCGCAGGTACGCTTCCTGCAATAG
GSP1	TCTATATTGTCAGTCGT
GSP2	AGAGCTTCTTTGTGGCTTGG
1334F	CCATGGCATTCTTGAGCGTTCTG
1334R	CATATGTAAATATCCTTTTTATTTATTATGG
1251F	ATCAGTGAGGCACCTGAGGCATCCAGTCAGTAATAG
1251R	CGGCGCGCCTCATGACTCTGACCAACTGAGCTATG
1252F	AAAAAGCTGAGATCTGCCTTACAAGCAGGATGTC
1252R	CCCCAGGCTTTACACGAGCCGGAAACGAAACTT
flaB-35F	TGATTAGCCTGCGCAATCATT
flaB-115R	AATGACAGATGAGGTTGTAGCAGC
bb0689-285F	AAGAGAAATACTGGCATCAGGAA
bb0689-362R	CTTCTTTGTGGCTTGGACTATTAAG
927F	AGAGGGAAATCGTGCGTGAC
927R	CAATAGTGATGACCTGGCCGT

^*a*^ Restriction enzymes sites are underlined.

### Complementation of the *bb0689* mutant

To generate a complementation construct, a suicide plasmid pOY928 was created. Specifically, the 1,058 bp downstream region of *bb0689* and the 1,372 bp DNA fragment comprising *bb0689* and its upstream regions were PCR amplified from OY444 using PCR primer pairs 1251F/1251R and 1252F/1252R, respectively. These two DNA fragments, together with the pUC Ori and the streptomycin-resistant cassette (i.e., PflgB-aadA) (62–64), were assembled together using the GeneArt Seamless Cloning and Assembly kit (Thermo Fisher Scientific). In the resultant plasmid pOY928, the PflgB-aadA was oriented in the opposite direction as *bb0689* transcription. Purified pOY928 was then transformed into the *bb0689* mutant using electroporation. Streptomycin-resistant transformants were selected and subjected to PCR and RT-PCR analyses to confirm that *bb0689* expression was restored in the complement strain.

### Expression of *bb0689* in *B. burgdorferi* infected mice

The murine needle-challenge model of Lyme borreliosis was used to examine *B. burgdorferi* infection in mammalian hosts (71, 72). When the growth of WT *B. burgdorferi* CE162 in BSK-II reached the mid-logarithmic phase (~5 × 10^7^ spirochetes/mL), spirochetes were standardized to 1 × 10^5^ spirochetes/mL using BSK-II. Groups of 3- to 4-week-old C3H/HeN mice (Charles River Laboratory) were intradermally inoculated with *B. burgdorferi* at a dose of 10,000 spirochetes (in 100 µL BSK-II medium) per mouse. At 11 days post-inoculation, mice were sacrificed, and heart, joint, skin from the injection site (i.e., injection skin), and skin from the distal back (i.e., distal skin) were collected. RNA was isolated and reverse transcribed to cDNA, and *bb0689* expression was quantified via absolute quantification qRT-PCR using the standard curve method.

### *In vivo* bioluminescence imaging

*In vivo* bioluminescent imaging was employed to decipher the role of *bb0689* in borrelial infection dynamics. Groups of C3H/HeN mice were injected intradermally with WT OY444, the *bb0689* mutant, or the complemented strain at a dose of 1 × 10^4^ spirochetes per animal. At 4, 6, 8, 10, 14, and 21 days postinfection, mice were intraperitoneally injected with D-Luciferin and then imaged using an IVIS Spectrum live animal imaging system at the Animal Imaging Core, University of South Florida. Bioluminescence imaging, image capture, luminescence quantification, regions of interest calculation, and data analysis were carried out as previously described (40–42, 73, 74). At the end of the experiment (i.e., day 21 postinfection), animals were euthanized, and tissues, including injection skin, distal skin, joints, and heart, were collected. Infection was determined as previously described (41, 64, 69, 75, 76). In brief, tissue samples were cultured for the recovery of motile spirochetes in BSK-II supplemented with an antibiotic mixture specific for *Borrelia* growth. A single growth-positive culture was used as the criterion for infection of each mouse.

### RNA isolation and qRT-PCR

Total RNA isolation from *B. burgdorferi* grown in BSK-II and from mice tissues, genomic DNA digestion, cDNA synthesis, RT-PCR, and qRT-PCR was performed as previously described (41, 62–64, 69, 75). For measuring gene expression using absolute quantification qRT-PCR, plasmids pOY928 and pOY16 were used as the template to generate the standard curve for *B. burgdorferi bb0689* and *flaB*, respectively. By using the standard-curve method, the copy numbers of *B. burgdorferi flaB* and *bb0689* were determined in each sample. Data are presented as the number of *bb0689* copies per 100 *flaB* copies. For gene expression analysis using relative quantification, the ΔΔC_T_ method was employed by using *flaB* as an endogenous control. Specific primers (*flaB*: flaB-35F/flaB-115R; *bb0689*, bb0689-285F/bb0689-362R) for qPCR are listed in Table 3.

### Rapid amplification of 5′ cDNA ends

5′ RACE was performed by using the 5’ RACE system, version 2.0 (Thermo Fisher Scientific) according to the manufacturer’s protocol. In brief, first strand cDNA was synthesized from total RNA using a *bb0689-*specific primer GSP1 (Table 3), and a homopolymeric tail was then added to the 3'-end of the cDNA using TdT (Terminal deoxynucleotidyl transferase) and dCTP. Tailed cDNA was amplified by PCR using GSP2 (Table 3) and the provided abridged anchor primer. Subsequent nested-PCR amplification was performed using GSP2 and the provided abridged universal amplification primer. The resulting product was ligated into pCR4-TOPO TA vector (Thermo Fisher Scientific) and transformed into *E. coli* TOP10. Purified plasmids were analyzed by Sanger sequencing to identify the transcriptional start site of *bb0689*.

### Luciferase reporter assay

To determine whether the 5′ regulatory region of *bb0689* contains a functional promoter, a fragment encompassing 303 bp sequences upstream of the *bb0689* ATG start codon was PCR amplified from *B. burgdorferi* using primer pair 1334F/1334R. Purified DNA was digested with NcoI and NdeI and then cloned into the promoterless *luc* reporter vector pOY63 (68). The resultant construct pOY976 was transformed into *B. burgdorferi* strain CE162 or the *rpoS* mutant by electroporation. *B. burgdorferi* was grown in BSK-II at 37°C with 5% CO_2_. When growth reached an appropriate growth phase, spirochetes were harvested by centrifugation and washed thrice with 1× PBS. Cell lysate preparations and luciferase assays were performed as previously described (41, 68, 77). Luciferase activity was measured using Biotek Synergy H4 hybrid microplate reader (Agilent Technologies), and the results were expressed as relative luciferase units per 10^6^ spirochetes.

### Sensitivity of *B. burgdorferi* to osmotic shock

To determine the tolerance of *B. burgdorferi* to high osmolarity, WT OY444, the *bb0689* mutant, and the complemented strain were grown in BSK-II at 37°C with 5% CO_2_. When growth reached the mid-logarithmic phase, NaCl was added to the culture to increase osmolarity by 1 M. Cultures were incubated at 37°C, and aliquots were removed at 0, 30, 60, 90, and 120 min. Spirochete viability was assayed by growth endpoint determinations as previously described (58, 63, 78). Percent survival was calculated as the number of viable spirochetes at each time point divided by the number of live spirochetes in the sample collected at 0 min.

### Statistical analyses

Unpaired *t* test or one- or two-way ANOVA, followed by Tukey test built in GraphPad Prism version 10.3.0 (GraphPad Software), was used to analyze data from different experimental groups for statistical differences. A *P*-value ≤ 0.05 was used to indicate statistical significance.

## Data Availability

The data that support the findings of this study are available from the corresponding author upon reasonable request.
